# Douglas L. Coleman, 1931–2014

**DOI:** 10.1007/s00125-014-3393-7

**Published:** 2014-10-07

**Authors:** Thomas R. Coleman

**Affiliations:** The Feinstein Institute for Medical Research, 350 Community Drive, Manhasset, NY 11030 USA

Early in the morning of 16 April 2014, my father, Douglas L. Coleman, died at his home in Maine, surrounded by family and friends. The cause of death was an erosive basal cell carcinoma that slowly, but inexorably, penetrated his face.

**ᅟ:**
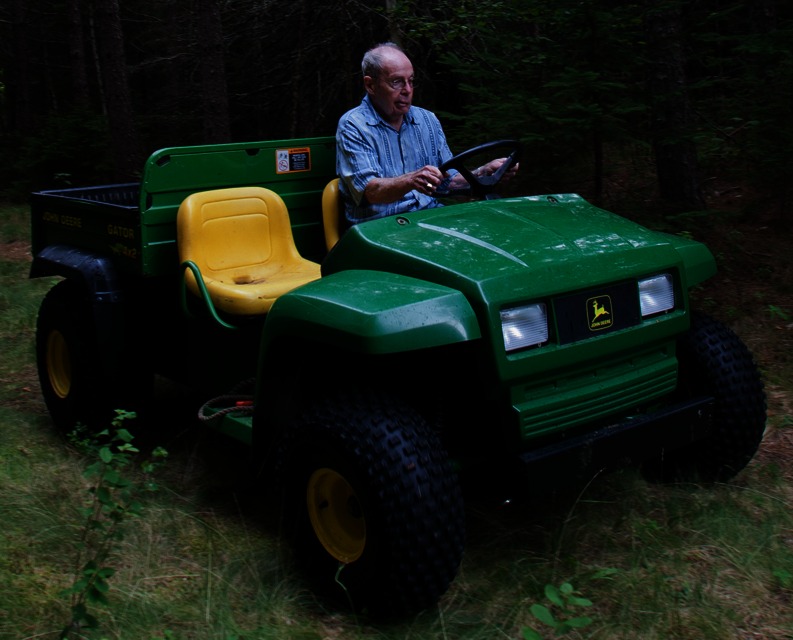
Photo by Françoise Gervais courtesy of The Jackson Laboratory

Dad was born in Stratford, Ontario, in 1931, an only child of impoverished parents. His family lived on cottontail rabbits, squirrels and the odd fish, mostly pike, supplemented with garden vegetables. Breakfast cereal was eaten dry, except on Sundays when he could have milk. Dad was the first Coleman to finish high school and he attended college at McMaster University in Hamilton, Ontario. It was at McMaster he met the love of his life and future wife, Beverly Benallick, the only female chemistry major. Dad attended graduate school at the University of Wisconsin in Madison where he pursued his interest in biochemistry in the laboratory of Professor Carl Baumann. When he received his PhD in 1958, the work prospects in Canada were not promising and he accepted a position at The Jackson Laboratory in Bar Harbor, Maine, where he spent his entire career.

In the 1960s, Dad became interested in two strains of obese mice that arose out of The Jackson Laboratory breeding stocks: *ob*/*ob* and *db*/*db*. While both of these mice strains are always hungry (hyperphagia) and consequently massively obese, the *ob* and *db* mutations are located on different chromosomes [1, 2]. Early experiments established that the diabetes resulted from a problem in the utilisation of insulin [3] and that the genetic background could have a profound effect on the severity of the disease [4]. Dad wondered whether some circulating factor might control the obesity. If such a hypothetical factor was present in the blood, he reasoned that he could test for its presence by linking the blood supplies of the animals. One such means to link blood supplies involved surgically joining two mice by skin-to-skin anastomosis—a technique termed parabiosis.

Dad’s parabiotic pairings were not only elegant but also led to the heretical conclusion that genetics could control obesity. In a control experiment, linking the blood supply of two normal mice, the pairs remained active and healthy for the duration of the experiment. In contrast, pairing *ob*/*ob* with normal mice caused the *ob*/*ob* mice to reduce their food intake to that observed in the normal–normal pairing [5]. The *ob*/*ob* mice, therefore, lost weight but survived for the duration of the experiment. This result suggested that the normal mice produce a blood-borne ‘satiety factor’ that could control obesity and that the *ob*/*ob* mice recognised and responded to this satiety factor.

Parabiotic pairings with the *db*/*db* mice were even more fascinating. When paired with normal mice, the *db*/*db* partners continued to gain weight, while the normal mice in each pair consistently died [6]. At necropsy, the normal mice lacked any food in their stomachs and had no glycogen in their livers: they had starved to death. Based on these results, Dad concluded that the *db*/*db* mice not only produced a blood-borne satiety factor powerful enough to starve a normal mouse but, in addition, were not responsive to this satiety factor themselves. The parabiotic pairing between *db*/*db* and *ob*/*ob* mice rounded out the analysis [5]. As before, the *db*/*db* pairs continued eating and gaining weight. In contrast, *ob*/*ob* pairs stopped eating, their blood sugar levels declined and they ultimately starved to death, albeit over a longer timeframe than the normal mice in a *db*/*db*–normal pair, due to a much more substantial adipose tissue reservoir in the *ob*/*ob* mice. Thus, the *ob*/*ob* mice, like the normal mice, responded to the factor provided by the *db*/*db* mice.

Taken together, the parabiosis experiments clearly indicated that the *ob*/*ob* mutant could respond to, but could not produce, a blood-borne satiety factor, while the *db*/*db* mutant overproduced a blood-borne satiety factor, but could not respond to it—perhaps owing to a lack of the necessary receptor. With the notable exception of receiving the Claude Bernard Award from the European Diabetes Federation in 1977 [7], these discoveries were largely dismissed at the time. Many in the obesity field favoured the leading thinking of the day: that mice (or people) are overweight because they overeat (behaviour) not because of a genetic predisposition (physiology). Although Dad correctly predicted that adipose tissue produced the factor, he was not able to identify the satiety factor as he focused his attention on isolating the wrong components (fatty acids and lipid extracts). Similarly, ventromedial nucleus lesions in *db*/*db* mice suggested that the primary defect in the *db*/*db* mice resided in the hypothalamus [8], but the satiety factor receptor would not be identified until decades later. In 1994, Dr Jeffrey Friedman and co-workers identified the satiety factor as a hormone, which they named leptin [9]. With the subsequent cloning of the leptin receptor [10] and the identification of the mutant receptor in the *db*/*db* mouse [11, 12], many predictions made based on the parabiosis experiments were proven: adipose tissue produced a satiety factor (leptin) that functions in a feedback loop to regulate appetite. The *ob*/*ob* mice lacked functional leptin but could respond to it; the *db*/*db* mice lacked functional leptin receptors and overproduced leptin in a futile effort to control their hunger. Dad was elected to the National Academy of Sciences in 1998 and received the Gairdner Foundation International Award (2005), the Shaw Prize (2009), the Albert Lasker Award for Basic Medical Research (2010), the King Faisal International Prize for Medicine (2013) and the BBVA Foundation Frontiers of Knowledge Award (2013).

Dad always felt that his career as a biochemist spanned the heyday of basic science. The NIH funding rate was >40%; The Jackson Laboratory charged 5% overheads. He enjoyed working independently to an extent that would be nearly impossible for a laboratory head to achieve today. His laboratory consisted primarily of himself and a single high school-educated technician that he employed for nearly three decades. Although he mentored one or two high school students each summer, he had only one graduate student and very few postdoctoral fellows during his career. I had never examined Dad’s curriculum vitae until I hosted him as a speaker at the Feinstein Institute for Medical Research (Manhasset, NY, USA) in 2009. When I did so, I was struck by the fact that a quarter of his publications were authored by only himself and the vast majority were authored by three or fewer researchers. Dad also felt that the public, through the NIH, paid for his research. As such, he believed that the public owned his discoveries and, therefore, like many scientists of his generation, he had mixed feelings about patenting scientific discoveries for personal gain. Indeed, partly motivated through this line of thinking, Dad donated all of his scientific prize money to various charities.

Faced with a changing research culture, Dad retired early in 1993. Unlike many emeritus scientists, he made a clean break, only returning to The Jackson Laboratory to use the fitness centre. To my delight, he entered retirement with great enthusiasm and spent his final years travelling and working on the town planning board as well as with forestry and conservation groups. While he will be remembered as a pioneer in demonstrating a genetic cause of obesity, he was very proud of the thousands of acres of Maine coastline and wetlands that he helped preserve for future generations.
